# Androgen Deprivation Therapy and Newly Developed Neovascular Age-Related Macular Degeneration Risk in Patients with Prostate Cancer

**DOI:** 10.3390/jcm13102978

**Published:** 2024-05-18

**Authors:** Jee Soo Ha, Do Kyung Kim, Hye Sun Lee, Soyoung Jeon, Jinhyung Jeon, Daeho Kim, June Seok Kim, Byeongseon Kim, Min Kim, Kang Su Cho

**Affiliations:** 1 Department of Urology, Prostate Cancer Center, Gangnam Severance Hospital, Yonsei University College of Medicine, Seoul 03722, Republic of Korea; eng.zsu@gmail.com (J.S.H.); dokyung80@yuhs.ac (D.K.K.); jun1644@yuhs.ac (J.J.); daehokim1@yuhs.ac (D.K.); idkjs12@yuhs.ac (J.S.K.); rusiawar92@yuhs.ac (B.K.); 2 Biostatistics Collaboration Unit, Yonsei University College of Medicine, Seoul 03722, Republic of Korea; hslee1@yuhs.ac (H.S.L.); jsy0331@yuhs.ac (S.J.); 3 Department of Ophthalmology, Institute of Vision Research, Gangnam Severance Hospital, Yonsei University College of Medicine, Seoul 03722, Republic of Korea; minkim76@yuhs.ac; 4 Center of Evidence Based Medicine, Institute of Convergence Science, Yonsei University, Seoul 03722, Republic of Korea

**Keywords:** wet macular degeneration, prostate neoplasms, testosterone

## Abstract

**Background/Objectives**: to evaluate the association between androgen deprivation therapy (ADT) and newly developed neovascular age-related macular degeneration (AMD) in patients with prostate cancer. **Methods**: We identified 228,803 men from the nationwide claims database in the Republic of Korea diagnosed with prostate cancer between 1 August 2009 and 31 December 2018 and followed until April 2021. Cases were defined as those newly diagnosed with neovascular AMD during follow-up. Cases were matched with controls based on age, index date, and follow-up duration, at a case-to-control ratio of 1:4. Adjusted odds ratios (aORs) of incident neovascular AMD associated with ADT were estimated using conditional logistic regression. **Results**: The main analysis included 1700 cases and 6800 controls, with a median follow-up of 3.42 years. ADT was associated with a reduced risk of incident neovascular AMD in patients with prostate cancer (aOR = 0.840; 95% confidence interval [CI], 0.743–0.951; *p* = 0.0058) in the multivariable analysis. A cumulative ADT duration less than 1 year was associated with a reduced risk of neovascular AMD (aOR = 0.727; 95% CI, 0.610–0.866; *p* = 0.0004); however, no association was observed when the duration of ADT was between 1 and 2 years (aOR = 0.862; 95% CI, 0.693–1.074; *p* = 0.1854) or more than 2 years (aOR = 1.009; 95% CI, 0.830–1.226; *p* = 0.9304). **Conclusions**: In patients with prostate cancer, medical castration for less than a year is associated with a reduced risk of incident neovascular AMD. These results suggest that androgens are involved in the pathogenesis of neovascular AMD.

## 1. Introduction

Prostate cancer, first described by Huggins and Hodges in 1941, is an androgen-dependent disease, and androgen deprivation therapy (ADT) plays an essential role in the treatment of patients with locally advanced, recurrent, and metastatic disease [1]. Castration can be achieved either medically or surgically; however, medical ADT using gonadotropin-releasing hormone (GnRH) agonist is the current mainstay of ADT [2]. The suppression of testosterone levels is associated with several adverse effects, such as insulin resistance, cardiovascular disease, osteoporosis, and fractures [3,4,5,6]. Since this hypogonadal status systemically affects the entire body, there has been an effort to understand the relationship between eye disease and testosterone suppression [7].

Age-related macular degeneration (AMD) is a common cause of blindness in elderly people in developed countries [8,9,10]. AMD is considered a disease continuum that progresses from early to late stages and can cause the progressive and permanent loss of central vision [11]. The development of AMD is influenced by multiple factors, including age, genetics, cigarette smoking, nutrition, diet, sunlight exposure, body mass index, and cardiovascular disease [12]. Neovascular AMD, also known as “wet AMD” or “exudative AMD”, is a late and serious type of AMD [13]. The introduction of anti-vascular endothelial growth factor (anti-VEGF) treatments has presented an opportunity to prevent blindness caused by neovascular AMD. Therefore, identifying and mitigating possible risk factors of neovascular AMD is critical for its successful management.

Some studies have shown no sex difference in the prevalence of neovascular AMD [10,14], while others have reported male predominance [15,16,17,18]. These results suggest that sex hormones may be involved in the pathogenesis of neovascular AMD. Because ADT suppresses serum testosterone levels to the level required for castration, we investigated the association between ADT and the incidence of neovascular AMD in patients with prostate cancer to elucidate the effect of changes in serum testosterone levels on the incidence of neovascular AMD.

## 2. Materials and Methods

### 2.1. Ethics

This study was performed in accordance with all applicable laws and regulations, good clinical practice, and ethical principles, as described in the Declaration of Helsinki. The Institutional Review Board of the Gangnam Severance Hospital approved the study protocol (approval number: 3-2021-0307). Patient-identifying information was not accessible from the Health Insurance Review and Assessment Service (HIRA) database.

### 2.2. Database

The National Health Insurance (NHI) System in the Republic of Korea is a universal health coverage system that covers more than 95% of residents in South Korea. The HIRA collects claims data submitted by healthcare providers for reimbursement and contains data on healthcare services for approximately 50 million beneficiaries each year [19].

### 2.3. Korean Rare Intractable Disease Copayment Program

South Korea has a special registration program for “rare intractable diseases” as declared by the Korean Ministry of Health and Welfare. “Rare intractable diseases” comprise 1123 diseases, including neovascular AMD, as of January 2022. Neovascular AMD has been designated as a “rare intractable diseases” since August 2009. Patients with neovascular AMD registered in this program are supported by NHI finances; therefore, their out-of-pocket medical cost is only a 10% deductible. To be registered in this program as having neovascular AMD (code V201), patients must have a confirmed diagnosis based on the NHI diagnostic criteria, as follows: (1) a dilated fundus examination using indirect ophthalmoscopy, (2) optical coherence tomography, and (3) fluorescein angiography to confirm neovascular AMD-related macular pathologic features. [20] Since the gold standard treatment for neovascular AMD requires expensive medications, such as ranibizumab, aflibercept, and bevacizumab, the HIRA verifies the accuracy and reliability of the diagnosis of neovascular AMD. Registration is specified by the V201 code, and all claims related to neovascular AMD include the V201 code along with the *International Classification of Diseases 10th Revision* (ICD-10) code for neovascular AMD (H35.31).

### 2.4. Study Cohorts

**Figure 1:**
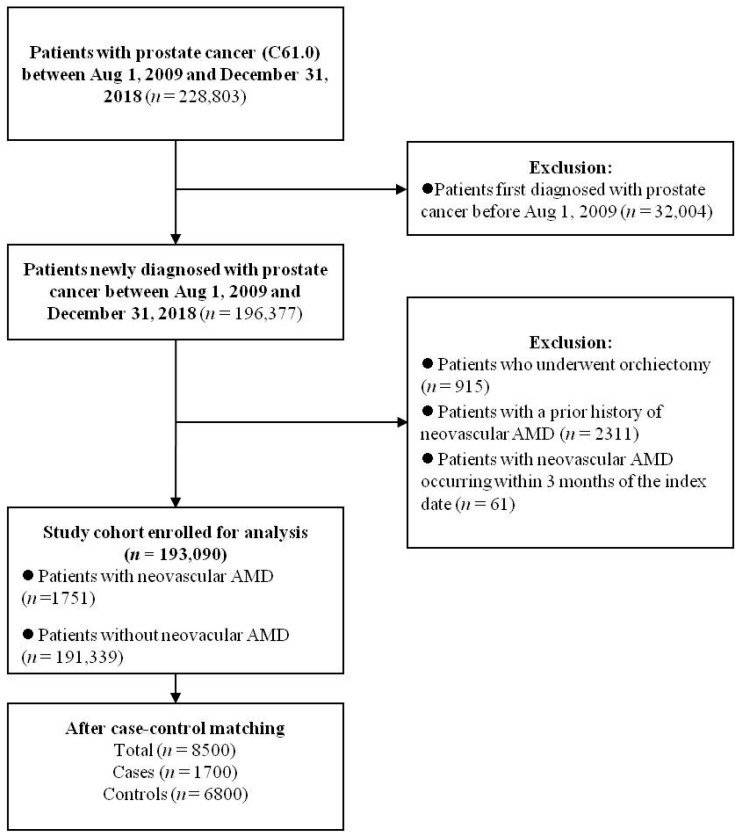
Flow diagram outlining the enrollment of the patient cohort. AMD, age-related macular degeneration.

ICD-10 codes were used to identify patients eligible for inclusion in this analysis. Considering the copayment program described above, the research period was set from 1 August 2009 to 31 December 2018. In total, 228,803 patients with prostate cancer (ICD-10 code: C61.0) were identified from the HIRA database. During the study period, 196,377 patients were newly diagnosed with prostate cancer. Patients who underwent bilateral orchiectomy were excluded (*n* = 915) because surgical castration accounted for an extremely small proportion of patients undergoing ADT in South Korea and we wanted to focus on the relationship between medical castration and neovascular AMD. Patients with a prior history of neovascular AMD (*n* = 2311) or neovascular AMD occurring within 3 months of the index date (see definition below) were also excluded (*n* = 61). Finally, the cohort consisted of 193,090 patients (Figure 1).

### 2.5. Nested Case–Control Study

A nested case–control study was conducted to investigate the association between the cumulative dose of ADT and newly developed neovascular AMD. A case was defined as a patient who visited a medical institution with a registration code of V201, which means that the patient was enrolled in a South Korean rare intractable diseases copayment program as a patient with neovascular AMD. A total of 1751 patients were identified. Cases were matched with controls based on age, index date, and follow-up duration, at a case-to-control ratio of 1:4.

### 2.6. Definition of Therapy Exposure and Covariates

The index date was set as the date of prostate cancer diagnosis. ADT was defined as at least one dose of a GnRH agonist or antagonist after the diagnosis of prostate cancer. The cumulative ADT duration was calculated as the sum of the action periods for each ADT preparation. When an event occurred during ADT treatment, the sum of the action periods of ADT preparation before the event occurred was set as the ADT duration. Covariates that may have a possible relationship with the incidence of neovascular AMD were identified using ICD-10 diagnostic codes at the index date (Supplementary Table S1). The covariates included patient age, cataracts, hypertension, diabetes mellitus, chronic liver disease, chronic kidney disease, cardiovascular disease, dyslipidemia, Parkinson’s disease, chronic pulmonary disease, myopia, vitreous floaters, and thyroid disease. We defined the end of the follow-up period as the date on which the event occurred or the date of the last valid inpatient or outpatient claim record.

### 2.7. Statistical Analysis

Statistical comparisons of continuous variables from patient baseline characteristics were performed using Student’s *t*-test, and categorical variables were compared using Pearson’s chi-squared test. Conditional logistic regression was used to estimate odds ratios (ORs) and 95% confidence intervals (CIs) for neovascular AMD in relation to ADT. We assessed whether there was a dose–response relationship between ADT duration and neovascular AMD. A sensitivity analysis was performed by repeating the same statistical procedure for 1:1 and 1:10 case-to-control ratios. *p* < 0.05 was considered statistically significant, and all statistical tests were two-sided. All study analyses were performed using the SAS^®^ System for Windows^®^, version 9.4 (SAS Institute Inc., Cary, NC, USA).

## 3. Results

The study cohort consisted of 193,090 patients newly diagnosed with prostate cancer between 1 August 2009, and 31 December 2018, with a median follow-up of 6.72 years (Figure 1). Baseline characteristics of the study cohort are presented in Table 1. Overall, 49,393 (25.6%) patients received ADT at some point during follow-up, whereas 143,697 (74.4%) did not. A total of 1751 patients were diagnosed with neovascular AMD. The main analysis included 1700 cases and 6800 controls, with a median follow-up of 3.42 years. The baseline characteristics after case–control matching are presented in Table 2. The cases and controls were properly matched.

ADT was associated with a reduced risk of incident neovascular AMD in patients with prostate cancer (adjusted OR [aOR] = 0.840; 95% CI, 0.743–0.951; *p* = 0.0058) in the multivariable analysis (Table 3). An accumulated duration of ADT less than 1 year was associated with a reduced risk of neovascular AMD (aOR = 0.727; 95% CI, 0.610–0.866; *p* = 0.0004); however, no association was observed for durations of ADT between 1 and 2 years (aOR = 0.862; 95% CI, 0.693–1.074; *p* = 0.1854) or more than 2 years (aOR = 1.009; 95% CI, 0.830–1.226; *p* = 0.9304).

As a sensitivity analysis to assess whether a case-to-control ratio of 1:4 was appropriate, 1:1 and 1:10 ratio age matching analyses were also performed (Supplementary Tables S2 and S3). In the 1:1 matching analysis, ADT was not associated with the risk of incident neovascular AMD (aOR = 0.885; 95% CI, 0.757–1.035; *p* = 0.1249), but a duration of ADT less than 1 year was associated with a reduced risk of incident neovascular AMD (aOR = 0.713; 95% CI, 0.576–0.883; *p* = 0.0019). In the 1:10 matching analysis, ADT was associated with a reduced risk of newly diagnosed neovascular AMD (aOR = 0.873; 95% CI, 0.776–0.982; *p* = 0.0242), and this association was most evident when the duration of ADT was less than 1 year (aOR = 0.785; 95% CI, 0.664–0.928; *p* = 0.0046). A 1:1 matching ratio prevented the loss of cases (cases = 1738; controls = 1738), whereas a 1:10 matching ratio allowed for an increase in the overall number of analyses (cases = 1639; controls = 16,390). The sensitivity analysis results showed a similar trend to that of the main analysis, indicating that a 1:4 matching ratio maintains both the case count and statistical power.

## 4. Discussion

There is a lack of sufficient research on the epidemiological characteristics of neovascular AMD, owing to its low incidence. A few limited cohort-based studies have reported the incidence of neovascular AMD; however, these studies were limited by having only a small number of cases of newly developed neovascular AMD, even though thousands of participants were included [9,18,21]. Park et al. demonstrated the nationwide prevalence and incidence rates of neovascular AMD (36.43 per 10,000 people and 3.01 per 10,000 person years in individuals 40 years of age or older, respectively) and male predominance (the male-to-female ratio of incidence was 1.61 in the general population and the male-to-female ratio of prevalence was 1.03) in the South Korean population [20]. Unlike previous HIRA database-based cohort studies that identified cases only using ICD-10 diagnostic codes, we identified neovascular AMD cases using the Korean rare intractable diseases copayment program registration code (V201), which is guaranteed by the NHI diagnostic criteria.

The effects of systemic hormonal changes on the development of AMD have been studied previously. Higher free thyroxine values, even in euthyroid individuals, were found to be an independent risk factor of AMD in a prospective population-based cohort study by Chaker et al. [22]. Similarly, Gopinath et al. demonstrated that overt hyperthyroidism (defined as serum thyroid stimulating hormone <0.1 mIU/L and free thyroxine >25.1 pM) was associated with an increased risk of incident AMD [23]. Although these studies are limited by the low incidence of AMD in the study participants and incomplete assessment of thyroid function, their results suggest that thyroid hormone might play a role in the development of AMD. The relationship between excess thyroid hormones and increased incidence of AMD can be explained by cumulative oxidative damage caused by the hypermetabolic state, and thyroid hormones can adversely influence retinal pigment epithelial cells [24,25]. The relationship between female sex hormones, such as estrogen, and the development of AMD has also been studied. Hwang et al. suggested that female reproductive factors were associated with the risk of neovascular AMD and that longer lifetime exposure to estrogen was also associated with a higher incidence of neovascular AMD in their cohort study, which enrolled postmenopausal women older than 50 years [26]. These studies show that systemic hormonal effects can influence the incidence of neovascular AMD. Therefore, investigating the effects of testosterone on neovascular AMD is a promising approach.

Androgens are essential for male sexual development and the functioning of the prostate gland. Extensive historical and contemporary data substantiate the role of androgens in the pathogenesis and progression of prostate cancer, referred to as the “androgen hypothesis [27]”. Huggins and Hodges suggested that the growth of prostate cancer was driven by androgens, based on their observation of the therapeutic effects of castration in patients with prostate cancer [28]. Androgens stimulate the initiation of tumors and the growth of xenografts in animal models, with tumor shrinkage observed following androgen deprivation [29]. However, despite the foundational scientific evidence endorsing the involvement of androgens in the development of prostate cancer, there exist contradictory clinical findings regarding the impact of endogenous testosterone levels on the initiation of human prostate cancer de novo [27]. Numerous longitudinal studies have confirmed a correlation between increased testosterone levels and the subsequent onset of prostate cancer [30,31,32].

Accumulating evidence has shown that sex hormones, such as estrogen and androgen, affect retinal disorders; however, the relationship between androgen and neovascular AMD is not conclusive yet [33]. Most clinical epidemiological studies examining the relationship between sex hormones and disease have included female participants, and for women, the role of female sex hormones can be inferred by comparing pre- and postmenopausal individuals. However, men do not experience events such as menopause in which the endogenous production of sex hormones abruptly and completely declines, making this type of comparative study difficult. ADT is a non-physiological method generally used for patients with locally advanced, recurred, and metastatic prostate cancer that completely suppresses serum testosterone levels. Depending on the disease burden of a patient with prostate cancer, ADT is administered either for a short period of less than 2 years or as lifetime maintenance therapy. Therefore, the effect of testosterone on the incidence of neovascular AMD might be revealed by investigating the development of AMD in patients undergoing different durations of ADT. A similar study was conducted using the Taiwan Longitudinal Health Insurance Database. Lin et al. demonstrated that patients with prostate cancer had an increased risk of AMD compared to patients without prostate cancer (adjusted hazard ratio [HR] = 1.25; 95% CI, 1.12–1.39; *p* < 0.001), and patients with prostate cancer who received ADT had a lower risk of AMD than those who did not (adjusted HR = 0.56; 95% CI, 0.41–0.76; *p* < 0.001) [34]. There are several potential reasons for this result. Prostate cancer progression is controlled by androgen receptor signaling and angiogenesis, which promote metastatic prostate cancer growth [35]. For prostate cancer, histopathology has identified elevated micro-vessel density and increased VEGF expression compared to non-neoplastic conditions [36]. Furthermore, VEGF levels correlate with more advanced tumor stages and grading, and plasma VEGF is elevated in metastatic prostate cancer compared to localized disease [37,38,39]. In a growing cancer, angiogenesis is induced by elevated expression of VEGF, which is regulated by the tumor microenvironment, including decreased oxygen levels and increased androgen levels [40,41]. Because the major clinical feature of neovascular AMD is choroidal neovascularization, there may be similar etiologies underlying neovascular AMD and cancer progression.

Our findings suggest that ADT is significantly associated with a reduced risk of neovascular AMD in prostate cancer patients (aOR = 0.882; 95% CI, 0.787–0.990; *p* = 0.0324). However, the association between neovascular AMD and duration of ADT is inconsistent. The administration of ADT for <1 year was associated with a reduced risk of neovascular AMD (aOR = 0.818; 95% CI, 0.694–0.964; *p* = 0.0166); however, no association was observed when the ADT duration was 1–2 years (aOR = 0.939; 95% CI, 0.765–1.153; *p* = 0.5484) or >2 years (aOR = 0.927; 95% CI, 0.779–1.104; *p* = 0.3944). These results imply that short-term ADT has a protective effect against neovascular AMD but that this association is duration-dependent. This duration-dependent difference can be explained as follows. First, the effect of androgens on the vascular system is time-dependent. Guo et al. reported increased hematocrit and whole-blood viscosity in the short term (2 weeks) in castrated male mice compared to that in normal male mice, but this effect was diminished in the long term (5–7 months), suggesting the presence of an adaptive mechanism [42]. Second, long-term ADT is associated with relatively advanced prostate cancer, which showed overexpression of VEGF [43]. In addition, oxidative stress occurs during the progression to castration-resistant prostate cancer, possibly masking the protective effect of ADT on neovascular AMD [44,45]. Third, administration of ADT can induce insulin resistance and metabolic syndrome, which are known risk factors of neovascular AMD [4,5]. This indirect side effect of ADT might occur after years of ADT.

This population-based study using data from the HIRA database has some limitations that should be addressed in future studies. Our database comprises the ICD-10 codes entered by physicians in South Korea. Serum testosterone levels and detailed results of the diagnostic method for neovascular AMD were not provided in the HIRA database. Furthermore, relevant lifestyle factors such as cigarette smoking, nutrition, body mass index, and other clinical data, which are known risk factors for neovascular AMD, were not available in this database. However, the Korean rare intractable diseases copayment program guarantees the diagnostic accuracy of neovascular AMD, and the advantage of using a large population-based cohort including the entire South Korean population cannot be denied. Differences in the incidence of neovascular AMD according to ethnicity have been reported in previous studies [46]. This study only targeted the South Korean population; therefore, additional research is needed to extend these findings to a broader population. To maintain a sufficient number of cases for reliable statistics, the control group was pairwise matched only for age, index date, and follow-up duration. In addition, since most medical ADT in South Korea during the study period involved GnRH agonists, the differences between GnRH agonists and antagonists could not be compared, even though it is known that there are different side effects depending on the type of medical ADT. For example, A significant decrease in the likelihood of cardiovascular events or mortality was observed with GnRH antagonists compared to GnRH agonists [47,48]. Additionally, GnRH antagonists exhibited markedly superior reductions in serum alkaline phosphatase levels compared to GnRH agonists, for which the impacts on bone density, bone strength, and fracture risk remains to be determined [49].

## 5. Conclusions

This study confirmed that medical castration for less than a year is associated with a decreased risk of neovascular AMD. This suggests that testosterone is involved in the pathogenesis of neovascular AMD. Our findings should be confirmed in a prospective study that assesses the pathophysiology of neovascular AMD in patients undergoing ADT for prostate cancer. Further research is needed on the correlation between the duration of ADT and neovascular AMD.

## Figures and Tables

**Table 1:** Demographic characteristics of the entire study cohort.

Variables	Overall (*n* = 193,090)	Case (*n* = 1751)	Control (*n* = 191,339)	*p* Value
ADT administration				0.0471
Never	143,697 (74.42)	1267 (72.36)	142,430 (74.44)	
Ever	49,393 (25.58)	484 (27.64)	48,909 (25.56)	
Cumulative duration of ADT				0.1664
None	143,697 (74.42)	1267 (72.36)	142,430 (74.44)	
<1 year	19,919 (10.32)	193 (11.02)	19,726 (10.31)	
1–2 years	11,618 (6.02)	123 (7.02)	11,495 (6.01)	
≥2 years	17,856 (9.25)	168 (9.59)	17,688 (9.24)	
Age, years	67.298 ± 10.258	71.026 ± 7.344	67.264 ± 10.275	<0.0001
Cataracts	69,794 (36.15)	823 (47.00)	68,971 (36.05)	<0.0001
Hypertension	86,146 (44.61)	773 (44.15)	85,373 (44.62)	0.6921
Diabetes	69,716 (36.11)	691 (39.46)	69,025 (36.07)	0.0033
Chronic liver disease	37,047 (19.19)	315 (17.99)	36,732 (19.20)	0.2014
Chronic kidney disease	11,772 (6.10)	108 (6.17)	11,664 (6.10)	0.9004
Cardiovascular disease	50,225 (26.01)	530 (30.27)	49,695 (25.97)	<0.0001
Dyslipidemia	89,944 (46.58)	819 (46.77)	89,125 (46.58)	0.8715
Parkinson’s disease	2468 (1.28)	27 (1.54)	2441 (1.28)	0.3235
Chronic pulmonary disease	81,216 (42.06)	782 (44.66)	80,434 (42.04)	0.0269
Myopia	16,291 (8.44)	138 (7.88)	16,153 (8.44)	0.4006
Vitreous floaters	4823 (2.50)	47 (2.68)	4776 (2.50)	0.6156
Thyroid disease	11,419 (5.91)	103 (5.88)	11,316 (5.91)	0.9553

Data are presented as *n* (%) or mean ± standard deviation. ADT, androgen deprivation therapy.

**Table 2:** Demographic characteristics after case–control matching.

Variables	Overall (*n* = 8500)	Case (*n* = 1700)	Control (*n* = 6800)	*p* Value
ADT administration				0.0034
Never	5896 (69.36)	1229 (72.29)	4667 (68.63)	
Ever	2604 (30.64)	471 (27.71)	2133 (31.37)	
Cumulative duration of ADT				0.003
None	5896 (69.36)	1229 (72.29)	4667 (68.63)	
<1 year	1177 (13.85)	191 (11.24)	986 (14.50)	
1–2 years	638 (7.51)	119 (7.00)	519 (7.63)	
≥2 years	789 (9.28)	161 (9.47)	628 (9.24)	
Age, years	70.960 ± 7.372	70.979 ± 7.386	70.955 ± 7.369	0.9046
Cataracts	3509 (41.28)	796 (46.82)	2713 (39.90)	<0.0001
Hypertension	3671 (43.19)	756 (44.47)	2915 (42.87)	0.2327
Diabetes	3200 (37.65)	676 (39.76)	2524 (37.12)	0.0439
Chronic liver disease	1466 (17.25)	308 (18.12)	1158 (17.03)	0.2881
Chronic kidney disease	551 (6.48)	105 (6.18)	446 (6.56)	0.5669
Cardiovascular disease	2384 (28.05)	516 (30.35)	1868 (27.47)	0.018
Dyslipidemia	3733 (43.92)	798 (46.94)	2935 (43.16)	0.005
Parkinson’s disease	106 (1.25)	27 (1.59)	79 (1.16)	0.1564
Chronic pulmonary disease	3680 (43.29)	760 (44.71)	2920 (42.94)	0.189
Myopia	594 (6.99)	135 (7.94)	459 (6.75)	0.0849
Vitreous floaters	176 (2.07)	46 (2.71)	130 (1.91)	0.0397
Thyroid disease	445 (5.24)	101 (5.94)	344 (5.06)	0.144

Data are presented as *n* (%) or mean ± standard deviation. ADT, androgen deprivation therapy.

**Table 3:** Relationship between ADT and newly developed neovascular AMD.

	Cases (*n* = 1700) *n* (%)	Controls (*n* = 6800) *n* (%)	Crude OR (95% CI)	*p* Value	Adjusted OR (95% CI) ^a^	*p* Value
**ADT administration**						
Never	1229 (72.29)	4667 (68.63)	Reference		Reference	
Ever	471 (27.71)	2133 (31.37)	0.827 (0.732–0.935)	0.0024	0.840 (0.743–0.951)	0.0058
**Cumulative duration of ADT**						
None	1229 (72.29)	4667 (68.63)	Reference		Reference	
<1 year	191 (11.24)	986 (14.50)	0.716 (0.602–0.852)	0.0002	0.727 (0.610–0.866)	0.0004
1–2 years	119 (7.00)	519 (7.63)	0.862 (0.693–1.072)	0.1809	0.862 (0.693–1.074)	0.1854
≥2 years	161 (9.47)	628 (9.24)	0.977 (0.805–1.185)	0.8125	1.009 (0.830–1.226)	0.9304

ADT = androgen deprivation therapy; AMD, age-related macular degeneration; OR, odds ratio; CI, = confidence interval. ^a^ adjusted for cataracts, hypertension, diabetes mellitus, chronic liver disease, chronic kidney disease, cardiovascular disease, dyslipidemia, Parkinson’s disease, chronic pulmonary disease, myopia, vitreous floaters, and thyroid disease.

## Data Availability

Data are contained within the article and Supplementary Materials.

## Supplementary Materials

The following supporting information can be downloaded at: https://www.mdpi.com/article/10.3390/jcm13102978/s1, Table S1: ICD-10 and billing codes for identification of diagnoses and medications; Table S2: Relationship between ADT and newly developed neovascular AMD (1:1 matching ratio); Table S3: Relationship between ADT and newly developed neovascular AMD (1:10 matching ratio).
