# Supplementary material for: Androgen Deprivation Therapy and Newly Developed Neovascular Age-Related Macular Degeneration Risk in Patients with Prostate Cancer
Source: J Clin Med. 2024 May 18;13(10):2978. doi: 10.3390/jcm13102978 (PMC11121844; doi:10.3390/jcm13102978)
Supplement: Supplementary file 1 [file jcm-13-02978-s001.zip › jcm-2930448-supplementary.pdf]

Supplementary Table S1. ICD-10 and billing codes for identification of diagnoses and medications

Comorbidity	ICD-10 code
Cataract	H25 (Senile cataract): H25.0, H25.1, H25.2, H25.8, H25.9<br>H26 (Other cataract): H26.0, H26.1, H26.2, H26.3, H26.4, H26.8, H26.9
Hypertension	I10 (Primary hypertension): I10.1, I10.9<br>I15 (Secondary hypertension): I15.0, I15.1, I15.2, I15.8, I15.9
Diabetes	E10 (Type 1 diabetes mellitus): E10.0, E10.1, E10.2, E10.3, E10.4, E10.5, E10.6, E10.7, E10.8, E10.9<br>E11 (Type 2 diabetes mellitus): E11.0, E11.1, E11.2, E11.3, E11.4, E11.5, E11.6, E11.7, E11.8, E11.9<br>E12 (Malnutrition-related diabetes mellitus): E12.0, E12.1, E12.2, E12.3, E12.4, E12.5, E12.6, E12.7, E12.8, E12.9<br>E13 (Other specified diabetes mellitus): E13.0, E13.1, E13.2, E13.3, E13.4, E13.5, E13.6, E13.7, E13.8, E13.9<br>E14 (Unspecified diabetes mellitus): E14.0, E14.1, E14.2, E14.3, E14.4, E14.5, E14.6, E14.7, E14.8, E14.9
Chronic liver disease	K70 (Alcoholic liver disease): K70.0, K70.1, K70.2, K70.3, K70.4, K70.9<br>K72 (Hepatic failure, NEC): K72.0, K72.1, K72.9<br>K73 (Chronic hepatitis): K73.0, K73.1, K73.2, K73.8, K73.9<br>K74 (Fibrosis and cirrhosis of liver): K74.0, K74.1, K74.2, K74.3, K74.4, K74.5, K74.6<br>B18 (Chronic viral hepatitis): B18.0, B18.1, B18.2, B18.8, B18.9
Chronic kidney disease	N18 (Chronic kidney disease): N18.1, N18.2, N18.3, N18.4, N18.5, N18.9<br>N03 (Chronic nephritic syndrome): N03.0, N03.1, N03.2, N03.3, N03.4, N03.5, N03.6, N03.7, N03.8, N03.9<br>N05 (Unspecified nephritic syndrome): N05.0, N05.1, N05.2, N05.3, N05.4, N05.5, N05.6, N05.7, N05.8, N05.9
Cardiovascular disease	I20 (Angina pectoris): I20.0, I20.1, I20.8, I20.9<br>I21 (Acute myocardial infarction): I21.0, I21.1, I21.2, I21.3, I21.4, I21.9<br>I22 (Subsequent myocardial infarction): I22.0, I22.1, I22.8, I22.9<br>I24 (Other acute ischemic heart disease): I24.0, I24.1, I24.8, I24.9<br>I25 (Chronic ischemic heart disease): I25.0, I25.1, I25.2, I25.3, I25.4, I25.5, I25.6, I25.8 I25.9<br>I50 (Heart failure): I50.0, I50.1, I50.9
Dyslipidemia	E78 (Disorders of lipoprotein metabolism and other lipidemia): E78.0, E78.1, E78.2, E78.4, E78.5
Parkinson's disease	G20 (Parkinson's disease)
Chronic pulmonary disease	J41 (Simple and mucopurulent chronic bronchitis): J41.0, J41.1, J41.8<br>J43 (Emphysema): J43.0, J43.1, J43.2, J43.8, J43.9<br>J44 (Other chronic obstructive pulmonary disease): J44.0, J44.1, J44.8, J44.9<br>J45 (Asthma): J45.0, J45.1, J45.8, J45.9<br>J47 (Bronchiectasis)

Myopia	H52.1 (Myopia)
Vitreous floaters	H43.38 (Other vitreous opacities)
Thyroid disease	E00 (Congenital iodine-deficiency syndrome): E00.0, E00.1, E00.2, E00.9<br>E03 (Other hypothyroidism): E03.0, E03.1, E03.2, E03.3, E03.4, E03.5, E03.8, E03.9<br>E05 (Thyrotoxicosis [hyperthyroidism]): E05.0, E05.1, E05.2, E05.3, E05.4, E05.5, E05.8, E05.9
**Medications**	**Billing Code**
GnRH agonists or antagonist	Degarelix: 624401BIJ, 624402BIJ<br>Goserelin acetate: 167202BIJ, 167201BIJ<br>Leuprorelin acetate: 182602BIJ, 182604BIJ, 182605BIJ, 182606BIJ, 182608BIJ, 182610BIJ, 182611BIJ<br>Triptorelin acetate: 244902BIJ, 467501BIJ, 467502BIJ
Anti-androgens	Bicalutamide: 117201ATB, 117202ATB<br>Cyproterone acetate: 139401ATB<br>Flutamide: 162101ATB

GnRH = gonadotropin-releasing hormone; ICD-10 = International Classification of Diseases, 10th revision; NEC = not else classified.

Supplementary Table S2. Relationship between ADT and newly developed neovascular AMD (1:1 matching ratio)

	Cases (n = 1738) n (%)	Controls (n = 1738) n (%)	Crude OR (95% CI)	P value	Adjusted OR (95% CI)[a]	P value
**ADT administration**						
Never	1256 (72.27)	1214 (69.85)	Reference		Reference	
Ever	482 (27.73)	524 (30.15)	0.879 (0.753-1.025)	0.0997	0.885 (0.757-1.035)	0.1249
**Cumulative duration of ADT**						
None	1256 (72.27)	1214 (69.85)	Reference		Reference	
<1 year	193 (11.10)	257 (14.79)	0.708 (0.573-0.875)	0.0014	0.713 (0.576-0.883)	0.0019
1-2 years	123 (7.08)	110 (6.33)	1.086 (0.820-1.439)	0.5648	1.072 (0.806-1.427)	0.6311
≥2 years	166 (9.55)	157 (9.03)	1.033 (0.807-1.321)	0.7983	1.057 (0.823-1.357)	0.6635

ADT = androgen deprivation therapy; AMD = age-related macular degeneration; OR = odds ratio; CI = confidence interval.

[a]adjusted for cataracts, hypertension, diabetes mellitus, chronic liver disease, chronic kidney disease, cardiovascular disease, dyslipidemia, Parkinson's disease, chronic pulmonary disease, myopia, vitreous floaters, and thyroid disease.

Supplementary Table S3. Relationship between ADT and newly developed neovascular AMD (1:10 matching ratio)

	Cases (n = 1639) n (%)	Controls (n = 16390) n (%)	Crude OR (95% CI)	P value	Adjusted OR (95% CI)[a]	P value
**ADT administration**						
Never	1182 (72.12)	11342 (69.20)	Reference		Reference	
Ever	457 (27.88)	5048 (30.80)	0.859 (0.763-0.966)	0.0109	0.873 (0.776-0.982)	0.0242
**Cumulative duration of ADT**						
None	1182 (72.12)	11342 (69.20)	Reference		Reference	
<1 year	188 (11.47)	2295 (14.00)	0.770 (0.652-0.910)	0.0021	0.785 (0.664-0.928)	0.0046
1-2 years	116 (7.08)	1224 (7.47)	0.904 (0.734-1.112)	0.3382	0.909 (0.738-1.119)	0.3698
≥2 years	153 (9.33)	1529 (9.33)	0.960 (0.798-1.156)	0.6676	0.981 (0.814-1.182)	0.838

ADT = androgen deprivation therapy; AMD = age-related macular degeneration; OR = odds ratio; CI = confidence interval.

[a]adjusted for cataracts, hypertension, diabetes mellitus, chronic liver disease, chronic kidney disease, cardiovascular disease, dyslipidemia, Parkinson's disease, chronic pulmonary disease, myopia, vitreous floaters, and thyroid disease.
